# Black henbane and its toxicity – a descriptive review

**Published:** 2014

**Authors:** Anahita Alizadeh, Mohammad Moshiri, Javad Alizadeh, Mahdi Balali-Mood

**Affiliations:** 1 *Pediatrician- Fellowship of clinical Toxicology Imam Reza Hospital, Mashhad University of Medical Science, Mashhad, I. R. Iran *; 2 *Department of Pharmacodynamy and Toxicology, School of Pharmacy, Mashhad University of Medical Science, Mashhad, I. R. Iran *; 3 *Health insurance research office, Armed forces insurance organization of Islamic Republic of Iran, Tehran, Iran (Mashhad branch)*; 4 *Mechanical Engineer, Hydro mechanical designer BS, Department of Dam and hydropower plant Hydromechanics of Tooss Ab Company, Mashhad, I. R. Iran*; 5 *Medical Toxicology Research Centre, Imam Reza Hospital, Faculty of Medicine, Mashhad University of Medical Sciences, Mashhad, I. R. Iran*

**Keywords:** *Black henbane*, *Hyoscyamus niger*, *Anticholinergic*, *Benzodiazepine*, *Poisoning*, *Physostigmine*, *Atropine*, *Hyoscyamine*

## Abstract

Black henbane (BH) or *Hyoscyamus niger*, has been used as a medicine since last centuries and has been described in all traditional medicines. It applies as a herbal medicine, but may induce intoxication accidentally or intentionally. All part of BH including leaves, seeds and roots contain some alkaloids such as Hyoscyamine, Atropine, Tropane and Scopolamine. BH has pharmacological effects like bronchodilating, antisecretory, urinary bladder relaxant, spasmolytic, hypnotic, hallucinogenic, pupil dilating, sedative and anti-diarrheal properties. Clinical manifestations of acute BH poisoning are very wide which include mydriasis, tachycardia, arrhythmia, agitation, convulsion and coma, dry mouth, thirst, slurred speech, difficulty speaking, dysphagia, warm flushed skin, pyrexia, nausea, vomiting, headache, blurred vision and photophobia, urinary retention, distension of the bladder, drowsiness, hyper reflexia, auditory, visual or tactile hallucinations, confusion, disorientation, delirium, aggressiveness, and combative behavior. The main treatment of BH intoxicated patients is supportive therapies including gastric emptying (not by Ipecac), administration of activated charcoal and benzodiazepines. Health care providers and physicians particularly emergency physicians and clinical toxicologists should know the nature, medical uses, clinical features, diagnosis and management of BH poisoning.

## Introduction

*Black Henbane* (BH) with the Latin name of *Hyoscyamus niger* is one of the common plants which may cause intoxication (Oztekin-Mat, 1994 ▶). Majority of the people (85%) who are interested in traditional medicines have used BH (Cirak et al., 2004 ▶). 

Exposure to potentially toxic plants like BH either intentionally or accidentally could induce toxic manifestations (Oztekin-Mat,1994 ▶; Fuchs et al., 2011 ▶; Moshiri et al., 2013 ▶). For example, Fuchs et al (2011) ▶ reported 42,193 cases of human plant exposure through 1995-2009. About 2.5% of calls (3872 poisoning cases) to Iranian Drug and Poisoning Information Centers in 2012 were made by poisonous plants (Ghane et al., 2013 ▶), and 11% of Norwegian Drug and Poisoning Information Centers inquiries in 2008 were about plants (Spillum and Muan, 2010 ▶). Although plant poisoning is rarely responsible for serious intoxication, they have the ability of lethal toxicity, especially in children (Fuchs et al., 2011 ▶).

Around 1.6% of 56,121 poisoned patients who referred to Poison Centre of Imam Reza of Mashhad of Iran, through 1981-1991, were accidentally poisoned by BH (Daneshvar et al., 1992 ▶ ).

Henbane is literally translated *“hen killer”* (Volak and Stodola, 1992 ▶), and its name is derived from the Anglo-Saxon Hen (chicken) and Bana (murderer), because when fowls eat its seeds, they become paralyze and die (Haas, 1995 ▶). However, the Greek’s root of Hyoscyamus (hyos=pig and cymos=bean) means Hog's-bean which animal is supposed to eat it with impunity (Volak and Stodola, 1992 ▶). BH is also named *Jupiter–bean, Syfonica, Cassilata, Cassilago, Caballinus, Henbell, Jusqiam, Bazrolbang, Mashe atar, Ajavainekhorasani, Devil’s eye, Stinking nightshade Insane root, Poison tobacco, Benele, Chenilsenkeraut* **(**Hocking, 1947 ▶). BH is classified scientifically into Angiosperms rake under Solanales order, Solanaceae family, Hyoscyamus genus and H. Niger species (Wikipedia, 2013 ▶).

Different forms of BH application include: an herb to produce herbal medicine, misused as a drug (abuse) and accidentally ingestion results in intoxication, especially by children. Even small amounts of all parts of the plant are able to induce intoxication; symptoms from dizziness to delirium along with other anticholinergic effects (Haas, 1995 ▶).

In this article, we will review the history, plant description, pharmacology, toxicology and case reports of BH intoxication.

**History**

BH has been used as a medicine since last centuries and the physicians in the past were more familiar with BH. Dioscorides (first century A.D.) had applied BH for treatment of sleeplessness and pain (Hocking, 1947 ▶). Pliny (first century A.D.) believed BH belongs to wine and reduces brain functions (Hocking,1947 ▶).The Greek ancients believed BH was the magical homeric nepenthes. Benedictus Crispus (681 A.D.) named it *Hyoscyamus* and *Symphonica*. Some virtues of BH have been recorded in the tenth century and it has been known as Jusquiasmus (the modern French name is Jusquiame). In Great Britain, hen thieves mixed the seeds of BH with grain and gave them to hens. The hens became dizzy and unconscious through night, when the thieves came to rub them (Paulsen, 2010 ▶). In the Norwegian tradition, women put an ointment contain mixture of some plants such as BH on parts of the body with thin skin and became hallucinated (Paulsen, 2010 ▶).

BH extraction was used by some witches to run or fly on fire (Paulsen, 2010 ▶) and also as "magic brews” (Carter, 2003 ▶). It Soothsayers applied BH as a component of their hallucinatory mixture (Lee, 2006 ▶). Moreover, BH has been used for criminal intoxication, for example in 1910 Dr Crippen poisoned his wife Cora, just before flying to United State with his mistress Ethel Le Neve (Lee, 2006 ▶; Anonymous, Wednesday, September 7th, 1910 ▶). It is believe that Hamlet’s father was killed by BH poisoning (Anonymous, 2013 ▶). A kind of necklace was made from BH root, as Anodyne necklace and dangled on neck of children to prevent convulsion and easy teething.

Interest of Anglo-Saxon (11th century) to herbal medicine resulted in attention to this plan again and they applied this plant for killing the worm of teeth as they believd) which induce toothache. BH was deleted from Britain pharmacopeia during 1746-1788 and was registered again in 1809. Baron Storch reintroduced it and he gave the extract of BH in cases of epilepsy and other nervous and convulsive stats. BH is cultivated in United State in17th century as a medicinal and ornamental plant (Pokorny and Mangold, 2010 ▶). BH was known in traditional medicine of china as *Tianxianzi *(Kirtikar and Basu, 1984 ▶; Li et al., 2011 ▶). In Indian and Chinese traditional medicine it was applied for treatment of stomachache, heavy cough, manic psychosis and neuralgic pains (Duke, 1985 ▶). Physicians of Tibetan used BH’s seeds as stomach pain reliever, anthelmintic, antitumor and febrifuge (Tsewang, 1994 ▶). Iranian physicians have named it Bazrolbanj or Banghdaneh (بذارالبنج ، بنگ دانه) and they applied it for several propose. They also used BH to abstinent therapy of opium addict persons (Nasiri et al., 2012 ▶). BH extract solution was topically administrated for earache and toothache (Hosseini Yekta and Sadeghpoor, 2012 ▶**). **BH was also recommended for treatment of chronic bronchitis, psychosomatic disorders, tremor, insomnia, neuropathic pain, abdominal pain and anti-convulsant (Kiasalar et al., 2007 ▶; Kiasalari, etal., 2011 ▶; Moradi et al , 2012 ▶). 

The L-hyoscine and some other active alkaloids of BH were isolated in the nineteenth century by Ladenburg (Lee, 2006 ▶). This compound has been applied in parasympathetic investigations.

**Plant description**

**Figure 1:**
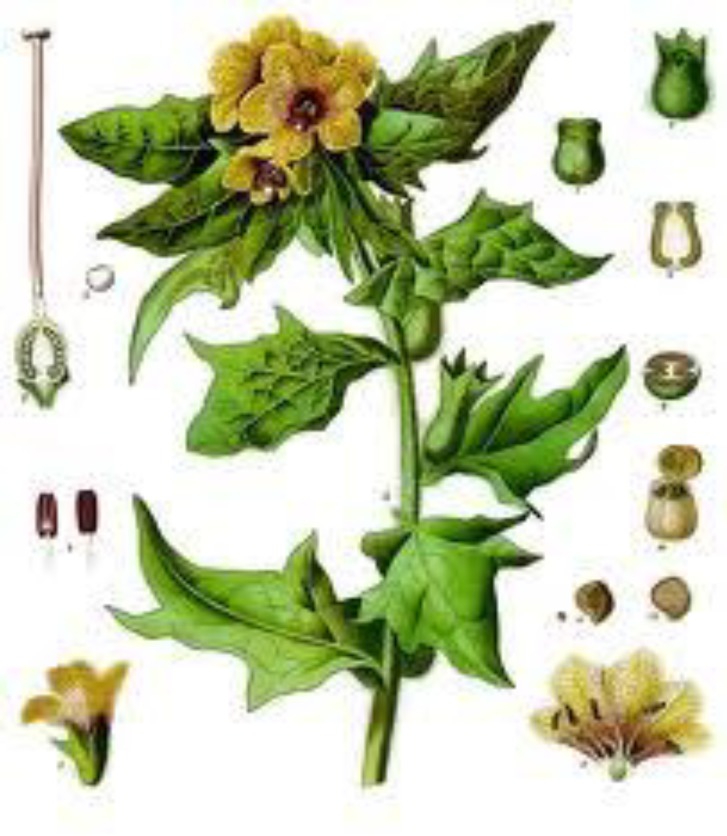
Black henbane

BH has considerable diversity of character as sometimes these variants are considered as different species (Grieve, 1913 ▶). BH is an annual or biennial and it grows up to three feet tall (Figure1).

The stems of mature BH are erect, leafy, branched, and densely covered with long glandular hairs and reaches 1 to 3 feet (Urkin et al., 1991 ▶; Daneshvar et al., 1992 ▶ ; Begum, 2010 ▶;Graham and Johnson, 2010 ▶). The leaves (cotyledons) are lance-shaped to oblong with a few hairs on the bottom (basal) margins (Graham and Johnson, 2010 ▶). The margins of leaves are slightly wavy. The leave veins are prominent and depressed on the upper surface (Graham and Johnson, 2010 ▶). This plant has a foul odor. Its taproot is thick and fleshy (Begum, 2010 ▶; Graham and Johnson, 2010 ▶). BH flowers are seen in June–September (Graham and Johnson, 2010 ▶), however the annual form flowers are in July or August and the biennial are in May and June (Grieve, 1913 ▶).The flowers are brownish-yellow and have a purple center and purple veins. They grow on long racemes in the axils of upper leaves (Graham and Johnson, 2010 ▶). 

Annual plant has shorter and weaker flowers than binaural form (Grieve, 1913 ▶). Hundreds of tiny black seeds, 1.5 millimeters long, are in egg-shaped fruit. As one plant produces about 10,000 seeds, the annual forms produce weaker and later developed seeds (Grieve, 1913 ▶; Begum, 2010 ▶; Graham and Johnson, 2010 ▶).

**Habitat**

BH is native to Europe and northern Africa (Pokorny and Mangold, 2010 ▶). It has been distributed in nearly all parts of north hemisphere, Europe, Asia, North America and Brazil (Grieve, 1913 ▶; Emami, 2007 ▶). There are about eleven species of the genus Hyoscyamus, distributed from the Canary Islands over Europe and Northern Africa to Asia. All of them have similar properties and ingredients. It is frequently found on chalky ground and particularly near the sea (Grieve, 1913 ▶). In Iran, this plant grows around Tehran, Karaj, North of Iran, Azerbaijan, Oroomie, Tabriz, Astara ,Ardabil ,Arak ,Tafresh, Roodbar, Gorgan, kordestan, khorasan, and some other areas (Daneshvar et al., 1992 ▶ ; Emami, 2007 ▶; Yousefi, 2007 ▶)

**Ingredients**

All part of BH, leaves, seeds and roots have been used or abused (Heber, 2004 ▶). BH contains some alkaloid and non alkaloid materials. Gas chromatography analysis of BH extraction has identified about thirty-four alkaloids (El Bazaoui et al., 2012 ▶).

Tropane alkaloids include Hyoscyamine, Atropine, Tropane and Scopolamine (Hyoscine), are present in BH as well as other Solanaceae (nightshade family) such as Atropa belladonna (deadly nightshade) and Daturaspp (thorn apples) (Li et al., 2006 ▶; Bernhoft, 2010 ▶; Paulsen, 2010 ▶). These compounds have different properties such as antispasmodic of smooth muscle, reduction of bronchial hypersecretion, and relief the gastric pain (Bernhoft, 2010 ▶).

Egyptian henbane has higher percentage of alkaloids (0.7-1.5%) than European strain (Begum, 2010 ▶). Atropine and scopolamine are more commonly found in leaves (Grieve, 1913 ▶; Robbers et al., 1996 ▶;Begum, 2010 ▶). Apoatropine (atropamine) and cuscohygrine are the main alkaloids of the root (Begum, 2010 ▶) and the main alkaloid of BH seeds are hyoscyamine and a little hyoscine and atropine (Grieve,1913 ▶; Uniyal, 1989 ▶). The seeds of this foul smelling plant have highest concentration of alkaloids (Frohne and Pfander, 1983 ▶; Graham and Johnson, 2010 ▶). The percentages of alkaloids of BH in the leaves, roots and seeds are in the order of around 0.17, 0.08 and 0.05 (Grieve, 1913 ▶; Frohne and Pfander, 1983 ▶; Begum, 2010 ▶). Some factors such as osmotic stress and microorganism injection influence on metabolic pathways of alkaloid synthesis in this plant (Ghorbanpour et al., 2010 ▶; Ghorbanpour et al., 2013 ▶). The alkaloids of all parts of BH will be toxic to animals and humans if they consume in large amount (Graham and Johnson, 2010 ▶). For example consumption of about 4 flowers of BH is sufficient to induce clinical manifestations in a pre-school child (Frohne and Pfander, 1983 ▶).

The two main alkaloid of BH; hyoscyamine and scopolamine, are used as medicines under controlled conditions. They applied as mild analgesic, antispasmodic, sedative and mydriatic (Begum et al., 2010 ▶; Pokorny and Mangold, 2010 ▶).

Hyoscyamine, secondary metabolite of BH, is levo-isomer of atropine, and has the same action with twice power of atropine. Hyoscyamine almost mixed with atropine (Ebadi, 2007 ▶). The major effect of Hyoscyamine is the central nervous system (CNS) depression (Frohne and Pfander, 1983 ▶; Cooper and Johnson, 1984 ▶). L-hyoscyamine is formed in the plant but it is readily hydrolyzed to atropine in the herbal cells and also in process of extraction (Begum et al., 2010 ▶; El Bazaoui et al., 2012 ▶). Atropine, which is (±)-hyoscyamine, has equal part of D and L hyosciamine (El Bazaoui et al., 2012 ▶). When atropine is hydrolyzed it forms (±)-tropic acid and tropine. Tropane alkaloids are derived from a combination of a piperidine and a pyrrolydine ring designated as a tropane (Zhang et al., 2007 ▶).

Furthermore BH has non tropane alkaloids such as calystegins that is a potent 

to moderate glycosidase inhibitor (Begum et al., 2010 ▶).

Although alkaloids have been known as the main cause of BH poisoning, its non-alkaloids components have not been well explored. The anticholinergic plants species also produces non-alkaloid secondary metabolites like withanolides, flavonoids, lignans, coumarinolignans, saponins, glycerides, glycosides and phenolics (Table 1) (Begum, 2010 ▶).

 The plant also has Non-alkaloidal compounds including hyosmin, Canabisin D, Canabisin G, Grossamide, Hyosciamide, Hyoscyamal Balanophonin, Cleomiscosin A, Cleomiscosin B, Hyosgerin, Rutin, Atroposide and Riboflavin (Grieve, 1913 ▶; Zhang et al., 2012 ▶)

BH seeds contain non alkaloids include: Lignans (Begum et al., 2009 ▶; Begum, 2010 ▶), coumarinolignans (Sajeli et al., 2006 ▶; Begum, 2010 ▶), lignanamides (Ma et al., 2002 ▶; Begum,2010 ▶), saponin (Begum, 2010 ▶), hyoscyamal, balanophonin, pongamoside D pongamoside C (Begum et al., 2009 ▶), and withnaloides (Ma et al., 1999 ▶; Begum, 2010 ▶). Begum et al (2010) ▶ have also found four coumarinolignans including cleomiscosin A, cleomiscosin B, cleomiscosin A-9'-acetate and cleomiscosin B-9'-acetate in methanolic extraction of BH seed. 

**Table 1:** List of Non-alkaloidal constituents isolated from *Hyoscyamus Niger *s*e*eds

**Secondary metabolite **	**Name of the compound**
**Lignans **	Hyosmin, Cannabisin D, Cannabisin G, Grossamide, Hyoscyamide, Hyoscyamal, Balanophonin
**Coomarinolignans**	Cleomiscosin A, Cleomiscosin B, Hyosgerin, Venkatasin, Cleomiscosin A, Methyl ether
**Withanolides**	Hyoscyamilactol, 16α-acetoxy-hyoscyamilactol, Daturalactone
**Glycerides**	1-* O* -octadecanoyl glycerol, *1- O - (9Z, *12Z-octadecadienoyl) glycerol, 1-* O* - (9Z, 12Z-octadecadienoyl) - 3 -* O* –nonadecanoyl glycerol. 1*- O-*(9z, 12Z-octadecadienoyl)–2-* O*-(12Z-octadecadicnoyl)glycerol, 1*- O - *(*9Z, *12Z-octadecadienoyl)-3 -*O*-(9Z-octadecanoyl) glycerol
**Flavonoids**	Rutin, Spiraeoside, 3', 5-Dihydroxy-3, 4', 5', 6, 7- pentamethoxyflavcm
**Flavonoids glyceride**	Pongamoside C. Pongamoside D
**Steroidal glycosides**	AtroposideA, Atroposide C, Atroposide E, Petunioside L
**Saponins**	Hyoscyamoside A, B, B1, B2. B3, C, CI, C2, D, D1 E, EI, F, FI, J and J1
**Phenolics**	Vanillic acid, Vanillin, Pinoresinol, *N*-trans- feruloyltyramine
**Miscellaneous**	5-(Hydroxymethyl) furfural, Daucosterol, β-sitosterol, 1, 24- tetracosanedioldiferulate, Riboflavin

**Pharmacological effects**

BH has bronchodilating, antisecretory, urinary bladder relaxant, spasmolytic, hypnotic, hallucinogenic, pupil dilating, sedative and anti-diarrheal properties (Gilani et al., 2008 ▶; Ghorbanpour et al., 2013 ▶).

Anticolinergic or parasympatholytic effect of BH relates to competitive inhibition of acetylcholine. This inhibitory effect is more prominent in muscarinic receptors than in nicotinic, ganglionic, or motor end plates receptors (Heber, 2004 ▶; Ebadi, 2007 ▶). However, there are some evidences that antispasmodic and selective airways and urinary bladder relaxant effects of BH extract of seeds (BHES) is not completely related to its anticholinergic property. BHES has shown a dose dependent relaxation on spontaneous contractions of rabbit jejunum, as well as verapamil. However, atropine has relaxed it partially. BHES has also relaxed guinea-pig trachea and rabbit urinary bladder which were constricted by carbachol, a cholinergic agonist (Gilani et al., 2008 ▶). BHES could shift the Ca concentration-response curves to right, the same as verapamil and dicyclomine. It seems that BHES has a Ca channel-blocking properties as well as anticholinergic effect (Gilani et al., 2008 ▶). Though the both fraction, organic and aqueous, have anticholinergic effect, only the organic part of BHES has shown Ca antagonist effect (Gilani et al., 2008 ▶). BHES has also shown the hypotensive, cardio-suppressant and vasodilator properties. It is believed that BHES lowers blood pressure through a Ca-antagonist mechanism and it is endothelium-independent (Khan and Gilani, 2008 ▶). 

BH has been traditionally used as anti-inflammatory drug and it is validated chemically and biologically (Begum et al., 2010 ▶). The methanolic extraction of BH seeds has shown acute and chronic analgesic and anti-inflammatory effects in animal models (Begum et al., 2010 ▶). It seems that cleomiscosin A is responsible for anti-inflammatory property of this extraction (Begum et al., 2010 ▶). 

The BH seeds have also shown antimicrobial, anti-diarrheal, antispasmodic and hypotensive effects (Khan et al., 1992 ▶; Begum, 2010 ▶). In addition, the methanolic extract of BH had anticonvulsant activity against picrotoxin-induced seizures in mice (Reza et al., 2009 ▶; Kiasalari et al., 2011 ▶).

 Parts of these properties are related to non alkaloids components (Khan et al., 1992 ▶; Begum,2010 ▶) . Liganamides, grossamide, cannabisin G and cannabisin D have shown cytotoxic effect in decreasing order (Ma et al., 2002 ▶; Li et al., 2011 ▶).

BH had been used as antiparkinson drug in Ayurveda. Methanolic extract of BH could significantly attenuate motor disabilities of parkinsonic mice. It may be also resulted from its monoamine oxidase inhibitory and hydroxyl radical scavenging potency as well as anticholinergic effect (Sengupta et al., 2011 ▶). Hydro-alcoholic extract of BH disarranges short-term memory of mice and reduces the learning of the water maze task (Hojjati et al., 2012 ▶).

Scopolamine (d-hyoscine), an important alkaloid of BH, acts similar to atropine as competitive antagonists of peripheral and central muscarinic cholinergic receptors (Brown and Taylor, 1996 ▶), but it passes off more quickly. As it is hypnotic, the pulse rate remains unchanged on therapeutic doses. Ophthalmic administration of scopolamine produces mydriasis more quickly than atropine with shorter duration. Scopolamine induces CNS depression, leading to drowsiness, amnesia and fatigue (Zaczek, 2001 ▶). Following to scopolamine administration, especially in large dose, a short stage of excitement and delirium with giddiness, uncertain movements, difficult and indistinct speech present and lead to sleep. The sleep usually is lasting 5-8 hours. Scopolamine also usually induces dry throat and thirst. The respiratory center does not influence as much as atropine. Atropine has lesser effect on CNS than scopolamine (Brown and Taylor,1996 ▶; Ebadi, 2007 ▶). 

However, scopolamine has shorter effect on peripheral nervous system than atropine and is able to depress the CNS in small doses as much as 0.5 mg (Ebadi, 2007 ▶). Although, the therapeutic doses of scopolamine are much lower than toxic doses, it is possible that scopolamine therapeutic doses induce toxic symptoms idiosyncratically (Ebadi, 2007 ▶).

Atropine, as an important alkaloid of BH, is white crystals, odorless, bitter taste, soluble in alcohol and chloroform (Zhang et al., 2007 ▶). 

It is applied as a premedication for anesthesia for bronchial secretions reduction and blocking bradycardia accompanied by some anesthetic drugs (Li et al., 2006 ▶). Atropine is an effective antimuscarinic agent for the treatment organophosphate compounds intoxication (Moshiri et al., 2012 ▶).

Atropine or atropinic drugs administration at therapeutic doses induce dry mouth, pupil dilation, inhibition of activity of sweat glands and, at toxic doses they are able to induce irritability, restlessness ,disorientation, hallucinations, delirium, tachycardia, palpitation, speech disturbance, and blurred vision(Hodgson, 2012 ▶; Balali-Mood et al., 2013 ▶ ; Etemad et al., 2013 ▶ ).

**Overdose**

*Clinical manifestations of poisoning*

 Due to high concentration of scopolamine in BH, ingestion of high dose of this plant primary leads to somnolence that followed by CNS excitation such as restlessness, hallucinations, delirium and manic episode. Livestock poisoned by BH present constipation and colic (in horses), dryness of the mucosa in the upper digestive and respiratory tract, pupil dilation (mydriasis), alterations in the heart rate and CNS effects like ataxia, irritability, restlessness, seizures and respiratory depression (Verstraete, 2010 ▶).

As mentioned above, all parts of BH are toxic and dryness or boiling could not destroy its alkaloids (Grieve, 1913 ▶). The most powerful part of BH is leaves, as even smell fresh leaves are able to produce giddiness and stupor (Grieve, 1913 ▶). Because of unpleasant odor and taste of BH leaves, animals avoid it and accidentally ingestion by human is rare, however, its root have occasionally been gathered and eaten (Grieve, 1913 ▶).

Intoxicated patients present symptoms like atropine overdose such as mydriasis, tachycardia, arrhythmia, agitation, convulsion and coma (Vidovic et al., 2005 ▶). Furthermore, BH intoxication could induce dry mouth, thirst, slurred speech, difficulty in speaking, dysphagia, warm flushed skin, pyrexia, nausea, vomiting, headache, blurred vision and photophobia, urinary retention, distension of the bladder, drowsiness, hyper-reflexia, auditory, visual or tactile hallucinations, confusion, disorientation, delirium, aggressiveness, and combative behavior (Schultes and Smith, 1976 ▶; Long et al., 1999 ▶; Pokorny and Mangold, 2010 ▶; Verstraete, 2010 ▶; Li et al., 2011 ▶; Anonymous, 2013 ▶; Prance and Nesbitt, 2005 ▶). 

Severe intoxication is accompanied with hypertension, respiratory arrest, coma and convulsions (Li et al., 2011 ▶; Anonymous, 2013 ▶).The manifestation such as hallucinations, restlessness, mydriasis and skin flushing are more common than the others. The skin irritation can be caused by contact to BH with bare skin (Pokorny and Mangold, 2010 ▶).

**Case reports and case series**

There are several case reports and case series on BH intoxication when it is ingested either accidentally or intentionally. We have summarized them as shown in Table 2. One of the most common manifestations of these cases is hallucination.

Dr. Stedman) 1751) reported 7 cases with delirium and hallucinations following henbane leaves ingestion. The patients imagined that everything around them were danger of falling. Three of them were not able to recognize their friends and were insensible. Another case report was a 34 year old woman who drank BH tincture. She suffered a burning sensation in her limbs that followed by losing their power through ten minute after ingestion. She also presented giddiness and intense thirst. The swollen purple rash especially on her face and neck also presented. She was numbed and unable to speak with a swollen tongue and dry mouth. Her pupils were dilated and at 7th hour after ingestion, her vision was limited and limb paresis was observed. After six days, she had been able to move her limbs although she lost her short-term memory and she could not remember what had happened (Anonymous, 2013 ▶). 

Gustav Schenk (1955) reported his experience of inhalation of BH‘s seeds fume. He described that he had great pain and physical discomfort within 15 minutes. Then, he suffered hallucination with marvelous thought such as separation of his body or talking his limbs with each other. 

Urkin et al (1991) ▶ reported 19 BH intoxicated cases during 1984-1989 that all of them were children in Bedouin. 

Fourteen cases were hospitalized in the autumn, 2 cases in summer and 3 children in spring. Reduced level of consciousness, restlessness (89.5%), hallucinations (89.5%), hot dry skin, and mydriasis (94.8%) were the most common manifestations Three children (15.8%) suffered deep coma. Other less common manifestations included: ataxia, involutionary movements, increased tendon reflexes, hypertension, tachycardia, vomiting, convulsions and hyperpyrexia. The children had been treated by intravenous physostigmine (36.8%) and sedatives such as diazepam and triclofos (31.6%). Most of the patients ingested the root of BH. Macchiaiolo et al (2010) ▶ reported a child with anisocoria following topical eye contact to his contaminated hand by Datura stramonium.

Daneshvar et al (1992) ▶ reported a case series, about 900 patients, that the majority of them were children with male dominant. Their cases had been most common admitted in spring (92%). The CNS stimulation was the main manifestation, of which hallucination and convulsion were the most common signs of intoxication. Turgul (1985) ▶ reported BH intoxication in 81.6% of 20 children who had eaten various parts of plants in Anatolia of Turkey during 1982 and 1983. The peak of intoxication was in May and June. He also revealed that 18.4% of intoxicated children had swallowed BH accidentally, 65.8 % of them had used it to produce a pleasant experience and 15.8 % had ingested just for trying. Some of them had tried it more than once. Nearly 85% of alkaloids were excreted within 24 hours. In another report, slurred speech (% 82.6) , aggressiveness ( 60.9%), dilatation of pupils (87%), flushing ( 87%) and somnolence ( 82%) were the most common manifestation of BH intoxicated in a group of children (Doneray et al., 2007 ▶).

All members of a family were intoxicated due to pull up a quantity of BH roots in soup. They were poisoned very fast with blurred vision, giddiness, sleepiness, delirium and convulsions (Grieve, 1913 ▶).

**Differential diagnosis**

**Figure 2:**
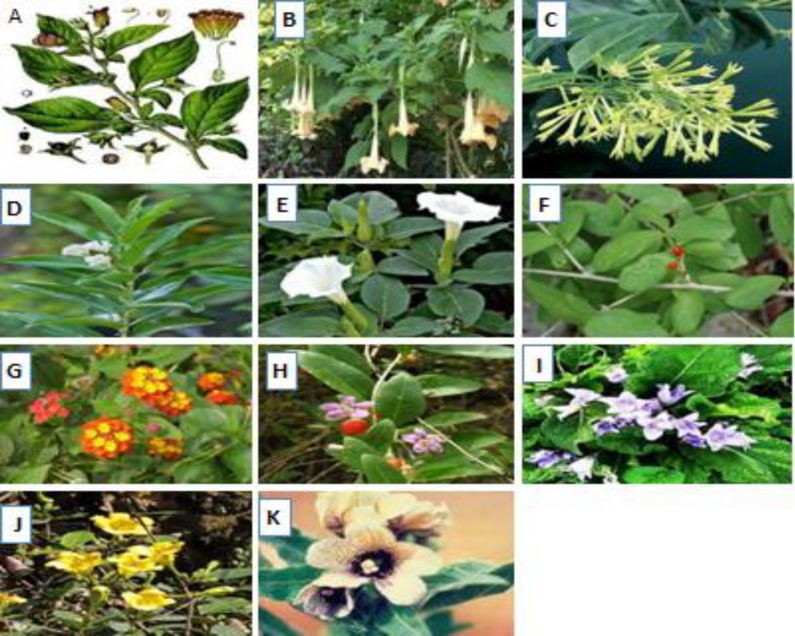
pictures of well-known plants that may cause anticholinergic syndrome in human toxicity.

Diagnosis of BH poisoning is based on history and clinical findings as previous mentioned, especially anticholinergic syndrome and hallucination (Vidovic et al., 2005 ▶; Doneray et al., 2007 ▶). There is some herbal and non herbal poisoning that should be considered for differential diagnoses of BH intoxication. Ingestion of some toxic plants that could induce anticholinergic syndrome, such as other members of Solanaceae family (*Datura stramonium and Atropa belladonna)*, are the most common poisoning cases for differential diagnosis; although they all are treated in same manner as BH poisoning. Morphological properties of some well-known plants that cause anticholinergic toxicity are shown in Figure 2. It should be considering that exact identifying the specific plant which causes anticholinergic toxicity is less important than the clinical findings, and treatment should be started even without of a positive botanical identification.

Numerous drugs are able to induce anticholinergic syndrome on therapeutic or toxic doses (table 4). Moreover, there are some critical medical conditions that might be mentioned for differential diagnosis such as meningitis, encephalitis, sepsis, head trauma, pheochromocytoma, heat stroke, CNS mass lesions, subdural hematoma, epidural hematoma, subarachnoid hemorrhage, cerebro-vascular accident, seizures with other etiology, psychosis and catatonia.

It is important for a physician to differentiate drug intoxication or the medical conditions from BH or other Solanaceae plants poisoning, because prognosis of intoxication by the drug intoxication is worse than poisoning by these plants. List of wellknown medicines that may cause anticholinergic syndrome in human beings, particularly after overdose was described in table 3. 

A: Atropa belladona, B: Brugmansia, C: Cestrum nactornum, D: Cestrum dirunum, E: Datura stramonium , F: Erythroxylum, G: Lantana spp H: Lyciumhalimifolium I: andragoraofficinarum, J: Solandra species k: Hyoscyamus Niger.

**Table 3:** List of well known medicines that may cause anticholinergic syndrome in human beings, particularly after overdose

**Drug class**	** Drug **
**Antihistamines**	Azatadine (Opimine), bromopheniramine (Dimetane®, Dimetap), chlorpheniramine (Chlor-Trimeton, Contac, Deconamine, Histussin, Naldecon, Tiraminic), clemestine (Tavist), carbinoxamine (Rondec), cyproheptadine (Periactin), dimenhydrinate (Dramaine), diphenhydramine (Benadryl®), hydroxyzine (Atarax, Vistaril), loratadine (Claritin, Reditabs), promethazine (Phenergan), pyrilamine (Triaminic), triprolidine (Actifed, Allercon)
**Antiparkinsonim**	Amantadine (Symmetrel), benzotropine (Cogentin), orphenadrine, trihexyphenidyl hydrochloride (Artane)
**Antipsychotics**	Chlorpromazine (Thorazine), droperidol (Inapsine), fluphenazine (Prolixin), haloperidol (Haldol), loxapine (Loxitane), mesoridazine (Serentil), molindone (Moban), perphenazine (Trilafon), pimozide (Orap), thioridazine (Mellaril®), trifluoperazine (Stelazine®), thiothixene (Navane)Antispasmodics (Gastrointestinal) Atropine, belladonna (Bellergal-S®), clindium bromide, dicyclomine hydrochloride (Bentyl®), Donnatol® (phenobarbital, atropine, hyoscyamine, scopolamine), glycopyrrolate (Robinul®), hyoscyamine (Levsin)
**Antispasmodics (Genitourinary)**	Flavoxate hydrochloride (Urispas®), Oxybutynin chloride (Ditropan®), Tolterodine (Detrol)
**Bronchodilators**	Ipratropium bromide (Atrovent)
**Carbamazepine (Tegretol)**	Carbamazepine
**Motion-sickness Medications**	Scopolamine, Meclizine (Antivert)
**Mushrooms**	Amanita species including muscaria, gemmata and pantherina Many other mushrooms species
**Ophthalmic Cycloplegics**	Cyclopentolate (Cyclogyl®), Homatropinehydrobromide, Tropicamide (Mydriacyl
**Skeletal Muscle Relaxants (central acting) **	Methocarbamol, , Metoxalen
**Cyclic antidepressants**	Amitriptyline ,Amitriptylinoxide, Butriptyline, Clomipramine , Demexiptiline, Desipramine, Dibenzepin, Dimetacrine, Dosulepin/Dothiepin ,Doxepin ,Imipramine, Imipraminoxide, Lofepramine, Melitracen, Metapramine, Nitroxazepine, Nortriptyline, Noxiptiline, Pipofezine, Propizepine, Protriptyline, Quinupramine
**Others**	Promethazine (Phenergan®) Cyclobenzaprine (Flexeril®), Orphenadrine (Norflex®)

**Treatment**

The main treatment of BH intoxicated patients is conservational. However, symptomatic and supportive therapies are the main objective (Daneshvar et al., 1992 ▶ ; Doneray et al., 2007 ▶). Gastric emptying is the first management of the patients after initial stabilization. The usual indications and contraindications, especially risk-benefit of this procedure, and suitable airway protection should be considering before gastric lavage (Vidovic et al., 2005 ▶; Gude and Hoegberg,2011 ▶; Li et al., 2011 ▶). Other notes include the amounts and time of ingestion, signs and symptoms of patients.

However, majority of authors have recommended gastric lavage for BH intoxicated patients (Daneshvar et al., 1992 ▶; Mofredj et al., 2000 ▶; Salen et al., 2003 ▶; Vidovic et al., 2005 ▶; Erkal et al., 2006 ▶; Doneray et al., 2007 ▶). Glatstein et al (2012) ▶ recommended applying gastric lavage up to 48 hour because of the inhibitory effect of anticholinergic materials on gastrointestinal motility. Salenet al (2003) ▶ evaluated the effect of gastric emptying on survival and length of hospital admission and need for intensive care or physostigmine therapy in 17 children who intoxicated with *Datura* *stramonium*, which is an anticholinergic plant belongs to a same family of BH. They found the seeds in only half of the patients’ gastric contents. They also revealed, in their retrospective study that removing the seeds had no effect on clinical endpoints of patients such as use of the ICU, length of staying in hospital or use of the physostigmine. As the risk of electrolytes imbalance secondary to gastric lavage is higher in children and the most of BH intoxicated cases are children, these complications should also be considered and treated accordingly (Mofredj et al., 2000 ▶ ; Gude and Hoegberg, 2011 ▶).

Activated charcoal is administrated considering its usual indications and contraindications. Another important supportive therapy of BH intoxicated patients is external cooling when hyperthermia presents. Psychomotor agitation of patients should be controlled as soon as possible with benzodiazepine. Physical restriction may prevent rhabdomyolysis and its secondary renal problems (Efstratiadis et al., 2007 ▶).

Benzodiazepines can be used against anxiety, restlessness and convulsion. Nonetheless they could not reversed delirium of patients admitted due to anticholinergic plant intoxication (Burns et al., 2000 ▶). Benzodiazepines have proposed as potential therapeutic agents for delirium (Lieberman, 2004 ▶). Large dose of benzodiazepine which could lead to over sedation, intensive care monitoring and respiratory support, may need to control of anticholinergic delirium (Koy, 2003 ▶).

Similar to some other anticholinergic poisonings, physostigmine as a specific antidote may be effective (Vidovic et al., 2005 ▶; Howland, 2011 ▶). It is especially recommended when tachycardia, somnolence, coma, and threatens respiratory arrest are developed (Li et al., 2011 ▶). It should be used with caution if there is suggested that patients have ingested tricyclic antidepressant (Pentel and Peterson,1980 ▶) or there is evidence of significant QRS or QT prolongation (Howland, 2011 ▶). Other precautions included the patients with past medical history of asthma, peptic ulcer disease, colitis, chronic obstructive pulmonary disease, cardiovascular disease (Koy, 2003 ▶). Physostigmine could pass blood brain barrier and thus effective against central anticholinergic symptoms (Glatstein et al., 2012 ▶). Frascogna (2007) ▶ believes that it is effective and safe drug in treatment of children with anticholinergic crisis for controlling agitation and reversing delirium. Recommended dose of physostigmine is 1 to 2 mg in adults and 0.02 mg/kg (maximum 0.5 mg) in children intravenously infused over at least 5 minutes. It could be repeated after 10 to 15 minutes if an adequate response is not achieved, however the onset of its action may occur within a few minutes (Frascogna, 2007 ▶; Howland, 2011 ▶).

 While the majority of authors recommend using physostigmine in treatment of anticholinergic plant intoxication, such as BH, there are some evidences that it may not be irrefutable therapy of these cases. Doneray et al (2007) ▶ reported 23 BH intoxicated children who have been treated without physostigmine in Turkey because it is unavailable in that country. None of them had any complications, and none required mechanical ventilation or died. All the children were discharged in good health throught 48 hours. In another retrospective study on *Daturastra ammonium *intoxicated patients, Salen and his colleagues (2003) ▶ revealed that physostigmine administration had no significant effect on survival, length of hospitalization and need for intensive care therapy (Salen et al., 2003 ▶ ).

On the other hand, Burns et al (2000) ▶ compared the effect of physotigmine and conservative benzodiazepine therapy on 52 referred patients for anticholinergic agitation and delirium, retrospectively. They found physostigmine had been able to control agitation more than benzodiazepine (96% versus 24% respectively). Anticholinergic delirium had been reversed by physotigmine but not by benzodiazepine (87% versus 0% respectively). Physotigmine administration, instead of benzodiazepine, had reduced CNS stimulation of poisoned patients. It is interesting that complications of physotigmine treated patients were much lower than the benzodiazepine treated (7% versus 46%). Nevertheless, they reported no significant difference on incidence of side effects and length of hospitalization between the two groups. The ineffectiveness of benzodiazepine on controlling agitation of patients with anticholinergic crisis has also been reported by Beaver and Gavin in 1998 ▶. Their patients had appropriate responses through 15 to 20 minutes to physotigmine (Beaver and Gavin, 1998 ▶). 

BH ingestion in pregnancy is not safe because atropine and other alkaloids readily pass the placenta and fetus is sensitive to tachycardia and hyperthermia. Ingestion of BH by patients with medical underling problems such as Down syndrome, Narrow-angle glaucoma, cardiac disease, pregnancy and breast-feeding needs more attention. BH poisoning has good prognosis and death from this plant toxicity is very rare.BH intoxicated patients referred to the poisoning ward of Mashhad Emam Reza hospital were discharged all in good health (Danshvar et al, 1992 ▶). However, Craig (1975) reported 8 fatalities in children due to antiocolonergic plant poisoning. One fatal case by Graev and Fallani (1960) ▶ and two by Tugrul (1985) ▶ were reported.

## Conclusion

There are many different plants in our environment and they have variable effects on human health. One of the most important and geographically common plants is Hyoscyamus Niger with therapeutic and possible toxic effects, which may be even fatal. Therefore, health professionals and medical care providers including physicians, particularly emergency physicians and clinical toxicologists should know the nature, medical uses, clinical features, differential diagnosis and management of BH poisoning.
